# Strain uses gap junctions to reverse stimulation of osteoblast proliferation by osteocytes

**DOI:** 10.1002/cbf.3245

**Published:** 2017-01-12

**Authors:** Rosemary F.L. Suswillo, Behzad Javaheri, Simon C.F. Rawlinson, Gary P. Dowthwaite, Lance E. Lanyon, Andrew A. Pitsillides

**Affiliations:** ^1^ Comparative Biomedical SciencesRoyal Veterinary CollegeLondonUK; ^2^ Institute of Dentistry, Barts & The London School of Medicine and DentistryQueen Mary University of LondonLondonUK; ^3^ School of Veterinary SciencesUniversity of BristolBristolUK

**Keywords:** coculture, gap junctions, mechanical strain, osteoblasts, osteocytes

## Abstract

Identifying mechanisms by which cells of the osteoblastic lineage communicate in vivo is complicated by the mineralised matrix that encases osteocytes, and thus, vital mechanoadaptive processes used to achieve load‐bearing integrity remain unresolved. We have used the coculture of immunomagnetically purified osteocytes and primary osteoblasts from both embryonic chick long bone and calvariae to examine these mechanisms. We exploited the fact that purified osteocytes are postmitotic to examine both their effect on proliferation of primary osteoblasts and the role of gap junctions in such communication. We found that chick long bone osteocytes significantly increased basal proliferation of primary osteoblasts derived from an identical source (tibiotarsi). Using a gap junction inhibitor, 18β‐glycyrrhetinic acid, we also demonstrated that this osteocyte‐related increase in osteoblast proliferation was not reliant on functional gap junctions. In contrast, osteocytes purified from calvarial bone failed to modify basal proliferation of primary osteoblast, but long bone osteocytes preserved their proproliferative action upon calvarial‐derived primary osteoblasts.

We also showed that coincubated purified osteocytes exerted a marked inhibitory action on mechanical strain–related increases in proliferation of primary osteoblasts and that this action was abrogated in the presence of a gap junction inhibitor. These data reveal regulatory differences between purified osteocytes derived from functionally distinct bones and provide evidence for 2 mechanisms by which purified osteocytes communicate with primary osteoblasts to coordinate their activity.

## 1 INTRODUCTION

There is much evidence supporting the view that osteocytes act as strain sensors in bones. Osteocytes with an extensive communication network and unique distribution are well situated within the bone matrix to sense mechanical loading and initiate a response by communicating with osteoblast and osteoclasts on bone surfaces.^1^, ^2^, ^3^, ^4^, ^5^, ^6^, ^7^, ^8^, ^9^, ^10^, ^11^, ^12^, ^13^, ^14^ Various studies have provided evidence to support this hypothesis. For example, it was shown that the level of intracellular glucose‐6‐phosphate dehydrogenase activity in resident osteocytes was influenced by, and related to, the level of exposure to mechanical loading in vivo.^15^ Moreover, within minutes following mechanical strain, osteocytes upregulate endothelial NO synthase and cyclooxygenase 2 expression, which in turn stimulate NO and prostaglandin E_2_ (PGE_2_) release, respectively.^16^, ^17^, ^18^ These are important early cellular changes that regulate downstream events including production of anabolic factors^19^ such as insulin‐like growth factor 1 and matrix molecules^20^ including dentin matrix protein 1. On the basis of such studies, it has frequently been proposed that osteocyte sensitivity to applied loads, sensed either as strain, flow, or some other sequelae of loading, provides the controlling input in the postulated “mechanostat,” which confers bone with its mechanoadaptive capacity.^21^, ^22^

Osteocytes are confined to lacunae, however, and can make little if any direct contribution to the architectural adaptive bone (re)modelling activities that load‐related strains might stimulate. It is assumed therefore that their influence is achieved via their control of the remodelling activity of osteoclasts (via osteoblasts and lining cells) and osteoblasts on the bone surface. A potential route by which osteocytes could influence the behaviour of overlying osteoblasts in response to external mechanical stimuli is via the passage of small molecules through the osteoblast: osteocyte network of gap junctions or via molecules secreted into the intralacunar fluid. This fluid bathes osteocytes and the bone‐facing processes of osteoblasts and lining cells, and its movement through canaliculae results from the pressure differentials induced by dynamic loads. The repetitive bending of the bone matrix is thought to generate a “pumping” action forcing fluid to the bone surface and subsequent dynamic shear strains on osteocytic processes.^10^, ^23^, ^24^, ^25^, ^26^, ^27^, ^28^

Previous studies have shown that gap junctions are expressed in all different types of bone cells^23^, ^24^, ^25^, ^26^, ^27^, ^28^, ^29^, ^30^, ^31^, ^32^, ^33^, ^34^, ^35^ and are likely candidates for chemical information transfer between bone cells,^36^, ^37^ providing evidence that gap junction communications are potentially important in mechanotransduction.^35^, ^37^, ^38^, ^39^, ^40^, ^41^, ^42^

Recent studies have examined the effects of fluid shear applied to an osteocytic cell line derived from long bone (MLO‐Y4)^35^, ^37^, ^41^, ^42^, ^43^, ^44^, ^45^, ^46^, ^47^ and shown that at least some consequences of this stimulation can be transmitted via gap junctions to otherwise unstimulated osteoblasts.^48^ Indeed, connexin 43 hemichannels have been postulated to serve a central function in fluid shear–induced PGE_2_ release from the MLO‐Y4 osteocytic cell line.^49^ Moreover, other studies have demonstrated that fluid flow increases the gap junction expression and function in the osteocytic MLO‐Y4 cells.^35^, ^41^, ^45^ In addition, mechanical stimulation also results in the opening of connexin 43 hemichannels and release of PGE_2_ 50 and adenosine triphosphate^46^ from the osteocytes.

Despite the attractive characteristics of these proposed models, there is, to our knowledge, little if any data that have demonstrated that purified osteocytes purified directly from different bones can exert any regulatory influence upon the behaviour of osteoblast. Furthermore, the mechanisms coordinating these interactions either under basal conditions or in response to mechanical strain also remain the subject of some speculation. In the present study, we have examined these osteocyte‐osteoblast interactions and the role of gap junction–mediated communication further. This has been achieved by investigating, for the first time, the influence exerted by purified embryonic chick bone osteocytes upon the proliferation of primary osteoblasts in direct contact coculture. We have also examined whether osteocyte‐osteoblast communication is modified by pharmacological blockade on functional gap junctions, both under these basal conditions and following application of mechanical strain in vitro. It has previously been shown that calvarial bone explants do not respond to mechanical loads,^51^ and so initial studies examined whether the source from which osteocytes were purified determined their influence upon primary osteoblast behaviour. Our studies reinforce differences between purified osteocytes derived from functionally distinct bones. In addition, they demonstrate that purified osteocytes can regulate behaviour of primary osteoblasts and that the functional outcome of this communication differs markedly when the proliferative response of osteoblasts to mechanical strain is examined.

## 2 MATERIALS AND METHODS

### 2.1 Cell isolation from embryonic chick bones

**Figure 1:**
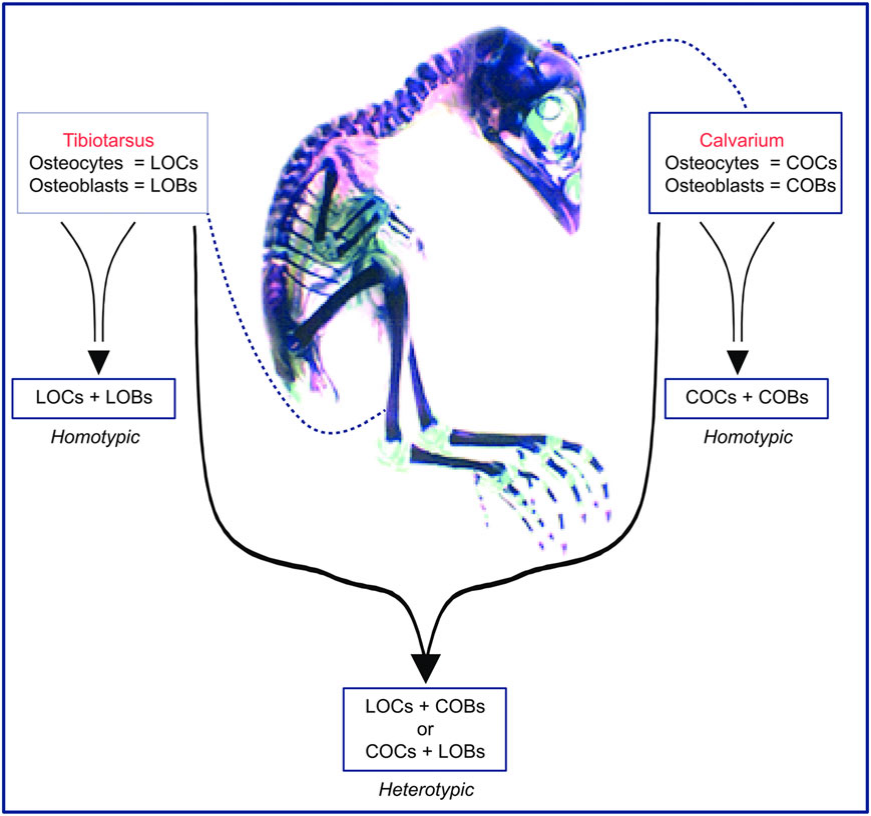
Experimental strategy used. Eighteen‐day‐old Alizarin Red S– and Alcian Blue GX–stained chick embryo depicting the harvest sites for both tibiotarsal and calvarial primary osteoblasts and osteocytes. Primary osteoblasts from both tibiotarsal long bone (LOBs) and calvariae (COBs) were either cultured alone or cocultured with osteocytes. The latter were also derived from both bone sites and were cocultured in either “homotypic” (LOCs + LOBs or COCs + COBs) or “heterotypic” (LOCs + COBs or COCs + LOBs) conditions

Purified osteocyte and primary osteoblasts were derived from both parietal bones and tibiotarsi using modification to methods used previously,^18^, ^52^, ^53^, ^54^, ^55^, ^56^ with primary osteoblasts derived and allowed to expand in culture prior to osteocyte purification. Briefly, parietal bones of the calvaria and tibiotarsal bones (see Figure 1) were removed from 18‐day‐old chick embryos and cleared of all attendant soft tissue and periostea. Medullary cavities were flushed with a Dulbecco phosphate‐buffered saline (lacking calcium and magnesium [PBS−]; Invitrogen, Paisley, UK), and resident bone cells were then dissociated using an adaptation to the method devised by van der Plas and Nijweide.^55^ This involved 3 sequential digestions of bone segments with 1‐mg/mL collagenase type 1 (*Clostridium histolyticum*, Sigma, Dorset, UK) in PBS^−^ followed by 4mM ethylenediaminetetraacetic acid (Sigma) in PBS^−^. Digestion was stopped by incubation with 10% heat‐inactivated chick serum in a Hank balanced salt solution (HBSS) (Invitrogen), and each of these 3 consecutive fractions was centrifuged (800 *g*, 4°C for 5 min) and then resuspended in heat‐inactivated chick serum in an HBSS on ice and combined, respun, and resuspended to a single‐cell suspension in PBS^−^ containing 4mM ethylenediaminetetraacetic acid and 0.5% bovine serum albumin (BSA; Fraction V, Sigma) (PEB) to produce a mixed bone‐derived cell population.

Osteocytes were purified from this mixed population using Ob7.3(5) mouse antichick osteocyte antibody as previously described (Nijweide, Leiden, the Netherlands).^52^ This involved addition of Ob7.3(5) to the cell suspension and incubation for 15 minutes on a “rotaspin” at 4°C. After centrifugation (800 *g*, 4°C for 5 min), cells were resuspended, washed in PEB, and incubated for 15 minutes at 4°C with MACS goat antimouse IgG microbeads (Miltenyi Biotech GmbH, Bergisch Gladbach, Germany). These cell suspensions were next passed through a prewashed mini‐MACS column with a magnetic collar (Miltenyi Biotech GmbH) and cells expressing epitopes bound by the Ob7.3(5) antibody (Ob7.3(5)^+^) and beads retained within the column, whilst other unbound cell types passed through to be collected (Ob7.3(5)^−^ cells). The column was washed with 5 volumes of PBS/BSA to remove all weakly bound, trapped, or otherwise unspecifically retained cells, and finally, removal of the mini‐MACS column from the magnetic field allowed Ob7.3(5)^+^ cells to be eluted. These methods achieved enrichment of chick osteocytes in samples immunomagnetically purified using monoclonal Ob7.3(5) antibody.

Enriched primary osteoblast cultures were obtained from the Ob7.3(5)^−^ cell preparation by immunomagnetic depletion of fibroblasts (using an antifibroblast antibody) to produce an osteoblast population of fibroblast‐depleted Ob7.3(5)^−^ cells. Using long bones and calvariae as primary sources therefore allowed Ob7.3(5)^−^ osteoblasts (LOBs and COBs) and Ob7.3(5)^+^ osteocytes (LOCs and COCs) to be separated.

### 2.2 Cell culture and characterisation

All bone‐derived cells were cultured in Dulbecco Modified Eagle Medium minus phenol red, 5% heat‐inactivated chick serum, 2mM l‐glutamine, 50 μg/mL gentamicin (Invitrogen); 50 μg/ml L‐ascorbic acid; 5.6 mM glucose (Sigma). Samples of each of the Ob7.3(5)^+^ and Ob7.3(5)^−^ cells had their phenotype confirmed by examination of morphology using scanning electron microscopy, immunocytochemical labelling with the Ob7.3(5) antibody, alkaline phosphatase activity, and in vitro mineralisation. For immunocytochemistry, cells seeded onto glass coverslips were incubated with 0.25% BSA in HBSS for 5 minutes to block nonspecific binding, incubated for 30 minutes at room temperature with Ob7.3(5) diluted 1:5 with BSA/HBSS, and washed and fixed in 4% buffered formaldehyde (VWR/Merck) in HBSS for 10 min at 4°C. The cells were then washed in 0.25% BSA in HBSS prior to 30‐minute incubation at room temperature in horse antimouse biotinylated secondary antibody (Vector Labs Limited, Peterborough, UK) (1:100 in BSA/PBS). After being washed, cells were incubated in the dark for 30 minutes at room temperature with streptavidin‐coupled CY‐3 (Vector, 1:500 in BSA/PBS), washed, and mounted in DAKO fluorescent mounting medium (DAKO Corporation, Carpinteria, CA, USA). Nuclear counterstaining was achieved using 4′‐6‐diamidino‐2‐phenylindole. Control samples were treated similarly but were incubated in the absence of either primary or secondary antibody.

Alkaline phosphatase activity was assessed using the Naphthol AS‐BI and Fast Blue BB method^57^ and was followed by counterstaining with a Meyer haemalum (VWR/Merck). Mineralisation potential was assessed with and without the addition of 50 μg/mL l‐ascorbic acid and 10mM β‐glycerophosphate (Sigma). These samples were reacted with alkaline phosphatase, counterstained with von Kossa and Safranin O (VWR/Merck), and visualised using an Olympus BH‐2 microscope.

### 2.3 Coculture and assessment of proliferation

Proliferation was assessed in both monoculture and in cocultures with cells seeded at a density of 20 000 total cells per well, in 24‐well plates (Nalge Nunc International). Preliminary experiments established an optimum osteocyte‐to‐osteoblast ratio of 4:1 and that primary osteoblasts should be allowed to adhere prior to the addition of osteocytes in coculture, prior to being allowed to settle overnight. After overnight serum depletion to synchronise proliferative activity, cultures were pulsed with methyl l[5‐^3^H]‐thymidine (1 μCi/mL per well, Amersham International, UK) and incubated for 18 hours. Rates of DNA synthesis were assessed by measuring [5‐^3^H]‐thymidine incorporation as previously described.^58^, ^59^ Briefly, cells were washed 3 times with ice‐cold PBS^−^, detached from the substrate with 0.25% trypsin (Sigma) and 100 μL of carrier DNA solution (1 μg of salmon sperm DNA; Sigma/1‐μL PBS) and 1 mL 10% trichloroacetic acid (TCA) added. The samples were vortexed and incubated for 16 hours at 4°C, and the TCA‐insoluble fraction was recovered by 3 sequential 1500 *g* centrifugations at 4°C, for 30 minutes, and washed with ice‐cold 5% TCA. The resultant pellet was dried with ice‐cold 90% ethanol and dissolved in a 20% formic acid (VWR/Merck) and 80% ACSII scintillant mixture (Amersham, Buckinghamshire, UK). Radioactive disintegrations per minute were counted using a 1214 Rackbeta liquid scintillation counter (LKB Wallac, London, UK). In experiments examining the effects of gap junction blockade, cells were incubated in a medium containing 20μM 18β‐glycyrrhetinic acid (Sigma).^60^

### 2.4 Application of mechanical strain

Mechanical strain was applied to cells seeded onto specially prepared (Nalge Nunc International, Naperville, Illinois) plastic cell culture–treated slides (75 × 25 mm), maintained in charcoal dextran (VWR/Merck) stripped medium and allowed to equilibrate in a humidified atmosphere of 95% air/5% CO_2_ after being placed in a custom‐designed jig, which loaded the strips in 4‐point bending.^4^, ^61^ Each strip was subjected to 600 cycles of applied load at 1 Hz, with each cycle producing a maximum longitudinal strain on the surface of the strip of 3000με. Control cells, on otherwise unperturbed slides, were treated identically but without strain stimulation. Following the period of strain, the slides were returned to plastic dishes, together with their surrounding medium and incubated for a further 18 hours after the addition of 1 μCi/mL of [^3^H]‐thymidine (an index of cell proliferation)^62^, ^63^, ^64^, ^65^, ^66^, ^67^, ^68^ to the medium.

### 2.5 Statistical analysis

Statistical analyses were performed using either Microsoft Excel or GraphPad Prism 6 (GraphPad Software, Inc., San Diego, California). Data are presented as mean ± SEM and were considered statistically significant when *P* ≤ .05. A 2‐sample, unpaired *t* test was used to compare means between control and treated groups.

## 3 RESULTS

### 3.1 Morphologically characteristic phenotypes retained in vitro in purified osteocytes

**Figure 2:**
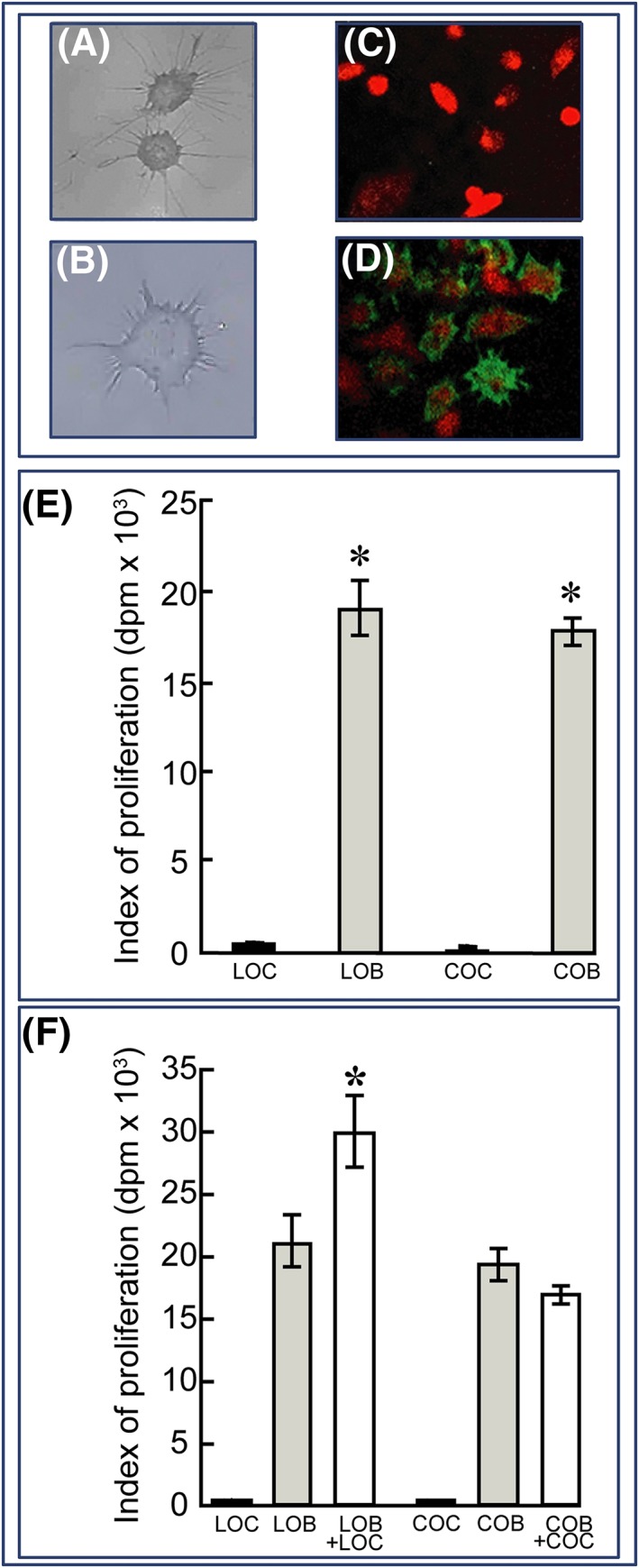
Calvarial and tibiotarsal osteocytes retain a morphologically characteristic phenotype. Ob7.3(5)^+^ LOCs and COCs populations (A and B) show the distinct stellate shape of the osteocytic phenotype. Immunocytochemical Ob7.3(5) antibody labelling of bone cells was assessed to ascertain population purity, with negative labelling in the fibroblast‐depleted Ob7.3(5)^−^ population (C) and positive staining in the Ob7.3(5)^+^ population (D). E, Proliferation potential of Ob7.3(5)^+^ and fibroblast‐depleted Ob7.3(5)^−^ populations was assessed by 18‐h pulse treatment with [^3^H]‐thymidine and incorporation. Both LOCs and COCs were shown to display negligible rates of proliferation. Conversely, LOBs and COBs proliferate avidly; disparity of proliferation allows for the study of osteoblast proliferation in mixed osteocyte‐osteoblast cultures. F, Homotypic cultures of Ob7.3(5)^+^ and fibroblast‐depleted Ob7.3(5)^−^ cells were pulse treated with [^3^H]‐thymidine. The presence of LOCs in the LOB cultures resulted in an increase of proliferation. Conversely, the presence of COCs in COB culture had no effect on COB proliferation rate. Data are presented to show incorporation of ^3^H‐thymidine over an 18‐h period, and all 6 wells of a 6‐well plate were used for each variable culture condition (*n* = 4 experiments in total). The asterisk denotes significance vs osteoblast monocultures (*P* < .05)

Using scanning electron microscopy, we found that Ob7.3(5)^+^ cells from both calvarial and tibiotarsal bones were generally smaller, exhibited a lower cytoplasmic area, had a distinct stellate appearance, and contained many more long slender processes radiating from a central cell body (Figure 2A and B). Furthermore, efficiency of purification and phenotypic stability were first confirmed by comparing Ob7.3(5) monoclonal antibody immunolabelling, which demonstrated negligible immunocytochemical labelling of isolated primary osteoblasts (Figure 2C), and unambiguous positive expression of the Ob7.3(5)‐directed epitope in osteocytes (Figure 2D and Table 1). This confirms the persistence of the osteocyte phenotype in Ob7.3(5)^+^ cells in vitro.

**Table 1:** Phenotype of Ob7.3(5)^+^ and fibroblast‐depleted Ob7.3(5)^−^ cell populations

Marker	Osteoblast	Osteocyte
Monoclonal antibody Ob7.3(5)	Negative	Positive
Proliferation	Positive	Negative
Alkaline phosphatase activity	Positive	Negative
Mineralisation	Positive	Negative

The fibroblast‐depleted Ob7.3(5)^−^ population displayed positive response for proliferation, alkaline phosphatase activity, and mineralisation reflecting an osteoblast phenotype. Ob7.3(5)^+^ cells were negative for these parameters.

Another characteristic of resident osteocytes is their postmitotic phenotype. To verify this postmitotic behaviour of immunomagnetically purified Ob7.3(5)^+^ cells, we compared their proliferation rates to Ob7.3(5)^−^ primary osteoblasts by measuring [^3^H]‐thymidine incorporation. We found that Ob7.3(5)^+^ purified from both calvariae and tibiotarsi showed negligible proliferation compared with Ob7.3(5)^−^ cells and that the latter showed similar rates of proliferation irrespective of whether they were sourced from tibiotarsi or calvariae (Figure 2E). Further distinction between Ob7.3(5)^+^ osteocytes and osteoblastic cells was confirmed by higher alkaline phosphatase activity and mineralisation potential in Ob7.3(5)^−^ cells and by a complete lack of such activities in Ob7.3(5)^+^ cells (Table 1). Henceforth, Ob7.3(5)^+^ osteocytes derived from calvarial or tibiotarsal long bones will be referred to as COC and LOC, respectively, and osteoblast‐like cells as COB (calvarial) and LOB (long bone).

### 3.2 Stimulation of primary osteoblast proliferation through coculture with long bone purified osteocytes

It is broadly held that osteocytes act as mediators of the changes in (re)modelling induced by load‐induced strain or fluid flow in vivo.^1^, ^2^, ^4^, ^5^, ^6^, ^7^, ^8^ Evidence for osteoblast regulation by purified osteocytes was therefore sought, and we found that the coculture of LOC with homogenic LOB significantly enhanced proliferation to levels greater than the expected sum attributable to osteoblasts and osteocytes cultured independently (LOB = 20 909; LOC = 394; LOB + LOC = 29 863 dpm; *P* ≤ .05; Figure 2F). In contrast, homogenic calvarial cocultures (COB + COC) did not show similar enhancement in proliferation (Figure 2F).

**Figure 3:**
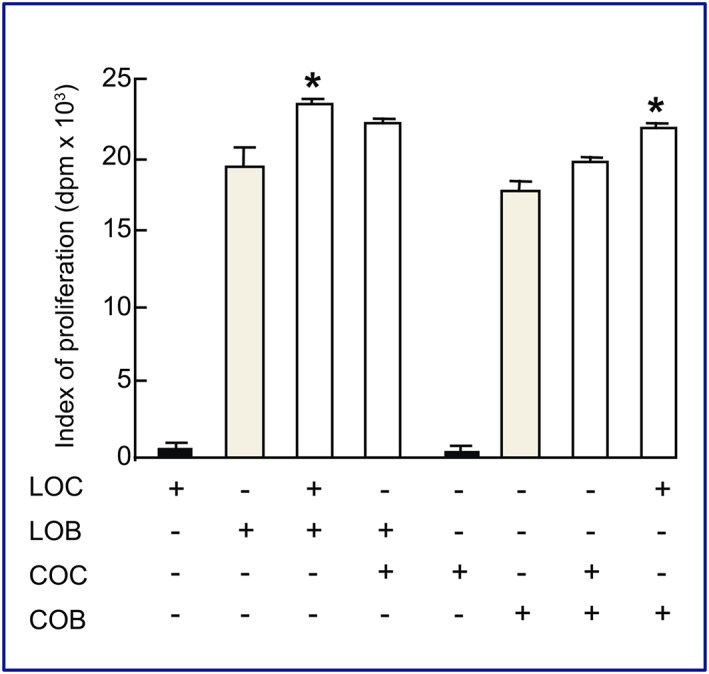
Long bone osteocytes enhance calvarial osteoblast proliferation. To assess whether COBs are unable to respond to osteocyte‐derived proliferative stimuli, COBs were maintained in heterotypic cultures with LOCs. LOCs induced proliferation of COBs, whilst COCs were unable to influence the proliferation of LOBs. This suggests the potential for fundamental differences signalling methods/signals derived from LOCs and COCs. Data are presented to show incorporation of ^3^H‐thymidine over an 18‐h period and all 6 wells of a 6‐well plate were used for each variable culture condition (*n* = 3 experiments in total). The asterisk denotes significance vs osteoblast monocultures (*P* < .05)

To address whether this osteocyte‐induced promotion of primary osteoblast proliferation is selective to purified osteocytes from long bones or an inherent characteristic of long bone osteoblasts, we also assessed proliferation rates in heterogenic cocultures (LOB + COC and COB + LOC). This showed that cocultures of COB with LOC showed significantly higher ^3^H‐thymidine incorporation levels than the sum of independent cultures (COB = 17 741; LOC = 554; COB + LOC = 22 065; *P* < .04; Figure 3). Such coculture‐related enhancement was not evident, however, when LOBs were cultured with heterogenic COCs (LOB = 19 073; COC = 109; LOB + COC = 21 719; *P* = .1; Figure 3), suggesting a selective influence of LOC on primary osteoblast proliferation. Together, these data indicate that osteocytes purified from long bones, but not those from calvariae, stimulate the basal proliferation of primary osteoblasts derived from either bone source.

### 3.3 Mechanical strain use of gap junctions to reverse proliferative influence of purified osteocytes on primary osteoblasts; gap junction–independent osteocyte‐related osteoblast proliferation

**Figure 4:**
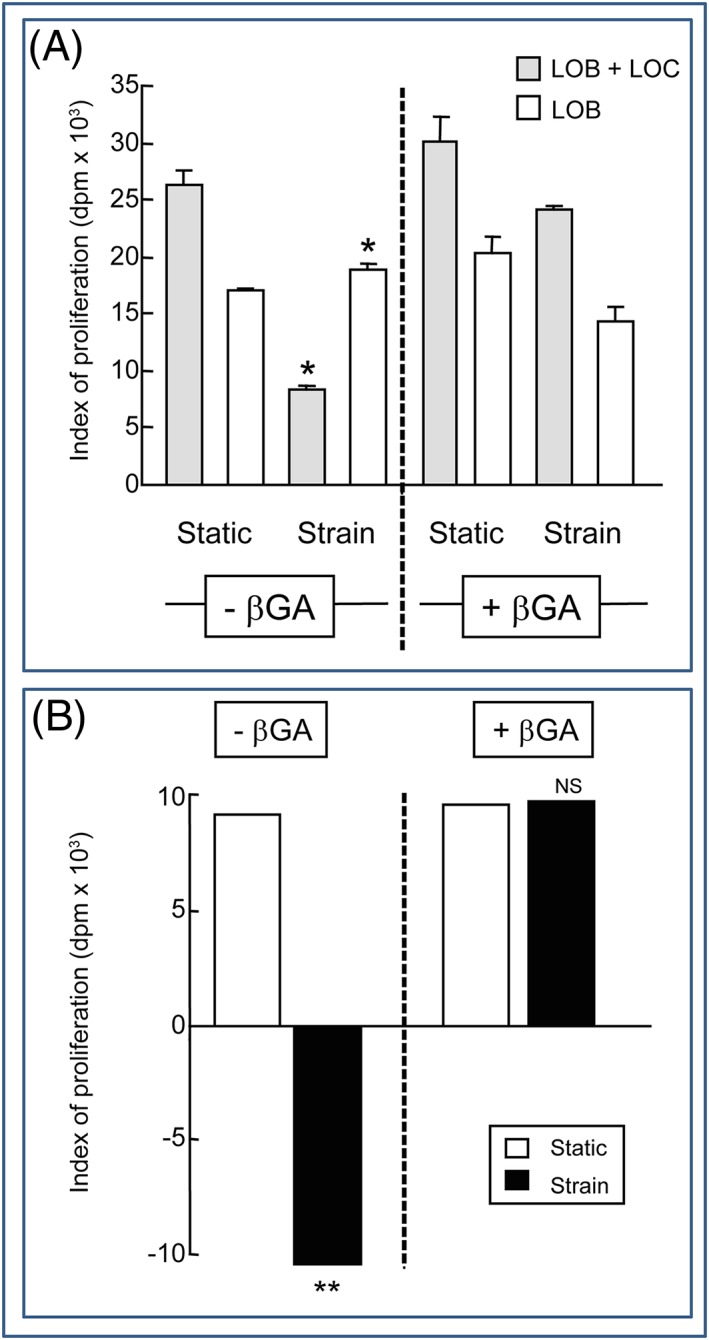
Proliferation in mechanically strained, not static, cultures is regulated by gap junctions. A, Mechanical strain increased LOBs proliferation in the absence of gap junction blocker. Somewhat surprisingly, the addition of LOCs in homotypic cultures resulted in a reduction in the proliferation rate following a period of mechanical straining. This effect was dependant on functional gap junctions as this was inhibited by the presence of the gap junction blocker, β‐glycyrrhetinic acid (β‐GA). B, Histogram showing the difference in osteocyte‐derived [^3^H]‐thymidine incorporation in data presented in A. These experiments identify differential methods of communication between osteocytes and osteoblasts in strained and nonstrained conditions. Data are presented to show incorporation of ^3^H‐thymidine over an 18‐h period, and 3 cell straining strips were used for each variable culture condition (*n* = 3 experiments in total; the asterisk denotes significance)

We found, consistent with previous studies, that mechanical strain application increases proliferation of primary osteoblasts cultured alone (Figure 4A). Surprisingly, however, we found that mechanical strain exposure evoked significant suppression of LOB proliferation when they were cocultured with LOC (Figure 4). To determine whether gap junction communication is required for LOC‐induced LOB proliferation and whether it contributes to bone cell responses to mechanical strain, we examined the effect of the gap junction blocker, β‐glycyrrhetinic acid (β‐GA), in homogenic long bone cocultures (LOC + LOB) both with and without mechanical strain stimulation. This revealed that LOC‐induced LOB proliferation in the absence of a mechanical strain stimulus was unaffected by β‐GA (Figure 4A) but, in contrast, that the mechanical strain–related suppression of osteocyte‐induced LOB proliferation was abrogated, and indeed reversed, in the presence of β‐GA (Figure 4B). Thus, treatment of mechanically strained cocultures (LOB + LOC + strain) with β‐GA results in similar levels of osteocyte‐induced proliferation as untreated, nonstrained LOC + LOB cocultures (Figure 4B). These data suggest that the influence of the purified osteocytes to regulating strain‐induced proliferation of primary osteoblasts is mediated by functional gap junctions but that their promotion of primary osteoblast proliferation in the absence of a strain stimulus is achieved independently of gap junction–mediated communication.

## 4 DISCUSSION

Monoculture systems are used most commonly to examine the mechanisms involved in bone cell biology. However, bone is an organ containing various cell types, and attempts to better replicate in vivo relationships have recently been shown to be pertinent.^69^ Osteocytes are considered as the mechanosensors of bone.^1^, ^2^, ^3^, ^4^, ^5^, ^6^, ^7^, ^8^, ^9^, ^10^, ^11^, ^12^, ^13^, ^14^ It is frequently asserted that they act to influence osteoblast (and osteoclast) behaviour to control remodelling activity to ensure mechanical competence, but evidence for this contention is sparse and unequivocal proof is lacking. A recent study performed in chick and mouse calvarial parietal bone shows, through FRAP analysis, the existence of cell‐to‐cell communication via gap junctions in the 3D morphology of the osteocyte network.^70^ Our use of primary osteoblast‐osteocyte cocultures, however, reveals additional relationships that would otherwise not be apparent in monoculture.

Using coculture, we sought evidence that purified osteocytes contribute to regulating primary osteoblast behaviour and whether any such contribution was reliant upon functional gap junctions. We found that postmitotic purified osteocytes were indeed capable of stimulating enhanced rates of proliferation by primary osteoblasts. Our data showed that osteocytes purified from mechanically responsive long bone, but not those from nonresponsive skull bones, exhibit a capacity to promote proliferation of primary osteoblasts. Using a pharmacological blocker (18β‐glycyrrhetinic acid) under these basal conditions, we also show that this osteocyte‐mediated promotion of proliferation of primary osteoblast was not dependent on functional gap junctions. Further to blocking gap junctions, 18β‐glycyrrhetinic acid has been reported to exert additional pharmacological actions including on 11β‐hydroxysteroid dehydrogenase 1, pannexin channel activity as well as high‐mobility group box protein 1 action, and glucocorticoid metabolism.^71^, ^72^, ^73^, ^74^ This is a limitation of our studies, and future examination of gap junction requires a more specific method of blocking these junctions. In marked contrast, we found that application of physiological levels of dynamic mechanical strain to cocultured long bone cells efficiently abrogated proliferation of primary osteoblast, with gap junction blockade indicating that strain‐related transfer of an inhibitory stimulus between purified osteocytes and primary osteoblasts involves functional communicating gap junctions.

The Ob7.3(5) antibody, which was used herein to isolate osteocytes, has been shown to be specific for the phosphate‐regulating gene with homology to endopeptidases on the X chromosome protein that is abundant in osteocytes.^52^ We find that cells isolated from chick bone (tibiotarsal or calvarial) using this antibody exhibit low, nonsignificant incorporation of [^3^H]‐thymidine when maintained in monoculture. This demonstrates their postmitotic characteristics and further supports the osteocyte specificity of the antibody and the purity of the resident cells isolated and used herein. Previous studies established and fully optimised this osteocyte immunomagnetic purification, and we, and others, have previously used these purified cells extensively to describe osteocyte in vitro behaviours.^18^, ^52^, ^53^, ^54^, ^55^, ^56^ Future characterisation using other markers including sclerostin and keratocan would provide additional evidence authenticating this cell population, but the phenotypic features and in vitro behaviour are consistent with terminally differentiated osteocytes. Use of MLO‐Y4 osteocyte‐like cell lines in our studies would have made it unfeasible to conduct our simple coculture studies as they, like primary osteoblasts, display avid proliferation in monoculture. Use of such a cell line, which was derived from one functional skeletal source (limb bone), would also make comparisons between osteocytes derived from functionally distinct calvarial bones impossible and may have obscured the specific influence that long bone–derived osteocytes exert on osteoblast behaviour. Indeed, the specificity of this influence is emphasised by the fact that osteocyte‐induced osteoblast proliferation is achieved in coculture only by osteocytes extracted from tibiotarsi and not by those extracted from calvariae and that osteoblasts derived from either source appear to behave similarly. These data not only rule out the possibility of some noncell, coculture effect but also pinpoint osteocytes derived from load‐responsive long bones rather than predominantly protective skull bones as distinct in their proproliferative osteoblast influence. They also suggest that the proliferative response to coculture with long bone–derived osteocytes is retained in primary osteoblasts derived from either bone site. The use of [^3^H]‐thymidine is widely reported as a method to assess proliferative capacity of bone cells^66^, ^67^, ^68^, ^75^; however, it is possible that cell survival is also affected. Although no evidence of an increased rate of cell death was observed in our experimental protocols, further studies to assess apoptosis directly would determine whether lack of proliferation might be associated with increased cell death.

The proproliferative influence of postmitotic osteocytes on cocultured osteoblasts is perhaps most readily interpreted, owing to the lack of apparent gap junction involvement, to suggest the involvement of an osteocyte‐derived soluble mediator. There are many candidate soluble mediators that are produced by osteocytes, which may account for this osteoblast stimulation. These include secretory phospholipase A_2_, which evokes increased PGE_2_ and prostaglandin I_2_ release from osteoblasts in nonloaded long bone organ explant cultures^5^ and factors such as transforming growth factor‐β and nitric oxide, which are produced by osteocytes.^76^, ^77^, ^78^ It is pertinent to emphasise that these proproliferative effects were limited to osteocytes derived from long bones but that those derived from calvariae, with distinct origins and protective rather than load‐bearing functions, had no effect on osteoblasts derived from either source. This is a fascinating and informative finding, suggesting differing physiological characteristics for osteocytes in bones with (parietal) and without (tibiotarsus) a cranial neural crest component. Data indicating that bone from these 2 distinct functional/embryological sites has distinct transcriptomes support this speculative conclusion.^79^

**Figure 5:**
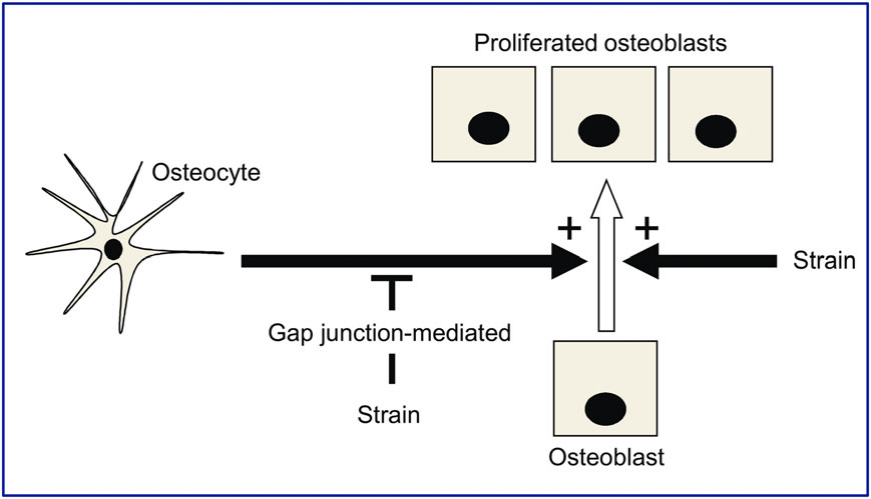
Schema depicting osteocyte‐derived signalling on osteoblasts. The results suggest that the proproliferative influence of osteocytes upon osteoblasts is reversed by the application of strain and that only this reversal is gap junction mediated

Few studies have used a coculture approach to study the signalling between bone cells, and fewer have incorporated an attempt to integrate mechanoresponse pathways. Organ culture models have been used with limited success.^5^, ^6^, ^80^, ^81^ Longer‐term cultures using MLO‐Y4 osteocytes and osteoblast‐like cell lines have established functional gap junctions between MLO‐Y4:MLO‐Y4 and MLO‐Y4:MC3T3‐E1 cultures.^37^ In a clever cell arrangement, MLO‐Y4s were mechanically stimulated without perturbing cocultured hFOB osteoblasts; osteoblast activity was regulated by functional gap junctions in response to osteocytes subjected to fluid shear strains.^48^ MLO‐Y4 osteocytes also influence osteoclastogenesis: In undisturbed cultures, MLO‐Y4 cells promote osteoclast formation,^82^ whereas mechanically stimulated MLO‐Y4 cells inhibit osteoclastogenesis,^83^ possibly via matrix extracellular phosphoglycoprotein.^84^ Our studies allow additions to the growing evidence that osteocytes can promote osteoblast proliferation, that this influence can be restricted by the application of strain, and that only the latter, osteocyte strain‐related control of osteoblast proliferation is dependent upon gap junctions (Figure 5).^69^

If our findings were to be directly extrapolated to the in vivo scenario, they would imply that, at rest, osteocytes act via transcellular signalling to maintain an active osteoblast population on the bone surface. Unlike the situation in culture, even at rest, osteocytes are constantly subjected to mechanical inputs, amongst which the most continuous is fluid shear strain stimuli driven by the circulatory system. In stark contrast, however, these proliferative signalling molecules emanating from osteocytes must rather be completely “overruled” in response to mechanical loading of bones, by information transferred to osteoblasts via gap junctions to promote an appropriate (re)modelling event. This implies that there is continual osteocyte‐derived signalling to cells on the bone surface maintaining the delicate balance of formation and resorption.

It is worth emphasising that the application of the mechanical strain stimulus is only transient in our studies, and yet this is nevertheless sufficient to significantly restrict the proliferation of osteoblasts induced by osteocytes. We interpret our findings to reflect the mechanism by which osteocytes and mechanical inputs together act to regulate the proliferation of osteoblasts. Thus, with increased loading, inhibition of proliferation would have to precede osteoblast differentiation at locations where new bone is required to withstand increased mechanical demands. An alternative interpretation is that osteocytes exert some hitherto unresolved suppression of the increases in proliferation that normally ensue periods of loading. In conclusion, our studies suggest that purified osteocytes, derived from load‐bearing long bones, exert a direct proproliferative influence upon primary osteoblasts and that mechanical strain may use gap junctions to reverse this osteocyte‐derived stimulatory effect.
